# Supplementary material for: Association between Plasma Omega-3 and Handgrip Strength According to Glycohemoglobin Levels in Older Adults: Results from NHANES 2011–2012
Source: Nutrients. 2022 Sep 29;14(19):4060. doi: 10.3390/nu14194060 (PMC9572262; doi:10.3390/nu14194060)
Supplement: Supplementary file 1 [file nutrients-14-04060-s001.zip › nutrients-1899712-supplementary.pdf]

Table S1. Types of ω-3 plasma fatty acids in individuals with elevated and normal glycohemoglobin. NHANES, 2011-2012.

ω-3 Plasma Fatty Acids	Total		Glycohemoglobin < 5.7%		Glycohemoglobin ≥ 5.7%	
	μmol/L	%	μmol/L	%	μmol/L	%
ALA	96.1	26.4	91.3	25.5	101.0	27.3
EPA	87.7	24.1	91.0	25.4	84.4	22.9
DHA	179.4	49.4	174.9	49.1	183.9	49.8
Total plasma ω-3 *	363.2	100	357.2	100	369.3	100

Notes: DHA: docosahexaenoic acid; EPA: eicosapentaenoic acid; ALA: alpha linolenic acid; * Total plasma ω-3 by the sum of ALA (18:3n-3), EPA (20:5n-3), DHA (22:6n-3).
